# Hydrogen-Bonded Di(hydroperoxy)alkane Adducts of the Type Cy_3_P=O·(HOO)_2_CHR (R = Alkyl)

**DOI:** 10.3390/molecules30020329

**Published:** 2025-01-15

**Authors:** Rahym Ashirov, Maya Todorovic, Nattamai Bhuvanesh, Janet Blümel

**Affiliations:** Department of Chemistry, Texas A&M University, College Station, TX 77842-3012, USA; rahymashirov@tamu.edu (R.A.); maya.todorovic@tamu.edu (M.T.); nbhuv@chem.tamu.edu (N.B.)

**Keywords:** phosphine oxides, di(hydroperoxy)alkanes, X-ray structures, multinuclear NMR, IR data

## Abstract

Five representatives of a novel type of di(hydroperoxy)alkane adducts of phosphine oxides have been synthesized and fully characterized, including their solubility in organic solvents. The phosphine oxide Cy_3_PO (**1**) has been used in combination with the corresponding aldehydes to create the adducts Cy_3_PO·(HOO)_2_CHCH_3_ (**2**), Cy_3_PO·(HOO)_2_CHCH_2_CH_3_ (**3**), Cy_3_PO·(HOO)_2_CH(CH_2_)_2_CH_3_ (**4**), Cy_3_PO·(HOO)_2_CH(CH_2_)_3_CH_3_ (**5**), and Cy_3_PO·(HOO)_2_CH(CH_2_)_7_CH_3_ (**6**). All adducts crystallize easily and contain the peroxide and phosphine oxide hydrogen-bonded in 1:1 ratios. The single crystal X-ray structures of **2**–**6** and their unique features are discussed. The ^31^P NMR spectra of the adducts **2**–**6** show downfield-shifted signals as compared to Cy_3_PO. In the IR spectra, the ν(P=O) wavenumbers of the adducts have smaller values than the neat phosphine oxide. All spectroscopic results of **2**–**6** show that the P=O bond is weakened by hydrogen-bonding to the di(hydroperoxy)alkane moieties. Adduct **6** selectively oxidizes PPh_3_ to OPPh_3_ within minutes, and nonanal is reformed in the process. The easy synthesis, handling, and administration of these stable, solid, and soluble peroxides with well-defined composition will have a positive impact on synthetic chemistry.

## 1. Introduction

### General Introduction

Hydrogen peroxide (H_2_O_2_) is an important and versatile oxidant used for diverse applications in industrial and academic settings [1,2,3]. However, H_2_O_2_ is commercially available only as dilute aqueous solution. Therefore, biphasic reactions are needed when reactants and products are only soluble in organic solvents that are not miscible with water. The latter may lead to secondary reactions and side-products. Aqueous H_2_O_2_ also decays at unpredictable rates and needs to be titrated prior to use [4]. Other types of peroxides have been applied, for example, urea hydrogen peroxide adducts [5,6,7], organic peroxocarbonates [8,9,10], peroxoborates [10], and organic peroxides [11]. However, these peroxides are not soluble in organic solvents, their composition is not stoichiometric, and, in the case of organic peroxides, there is a safety issue. Adducts of H_2_O_2_ with organic solvents are known, but they also pose safety risks, and are not shelf-stable, and have to be prepared immediately before being applied as oxidizers [12,13,14,15].

Superior, alternative solid adducts of peroxides have been sought. Phosphine oxides can act as electron pair donors for diverse HO groups and form strong hydrogen bonds [16,17,18,19,20]. Indeed, phosphine oxides could successfully be used to stabilize H_2_O_2_ and di(hydroperoxy)alkanes, and they facilitate crystallization [21,22,23,24,25,26,27,28,29]. While phosphines are ubiquitous as ligands and play crucial roles in catalysis [30,31,32,33,34], phosphine oxides are less prominent. However, they are important for characterizing surfaces [35,36,37,38,39] and as synthetic targets [40,41,42,43,44]. Phosphine oxides have been used previously for Mitsunobu reactions [45,46] and recently in redox-free Mitsunobu organocatalysis [47].

All of the phosphine oxide adducts of the type R_3_PO∙(HOO)_2_CR′R″ (R = alkyl, aryl) are shelf-stable over months, their composition is well-defined, they have high melting points, and they are soluble in organic solvents. These adducts have been successfully employed in diverse reactions, e.g., selective phosphine oxidation, sulfide to sulfoxide oxidation, the direct oxidative esterification of aldehydes, and Baeyer–Villiger oxidations [21,22,23,24,25,26,27,28,29].

In this contribution, we describe a hitherto unrealized type of peroxide adduct of phosphine oxides in order to probe and expand the range of possible adducts. From a practical point of view, we sought to reduce the “dead weight” of the adducts to render them more competitive with commercial aqueous H_2_O_2_ while retaining their oxidative power. Therefore, the new adducts Cy_3_PO∙(HOO)_2_CHR (R = alkyl) are derived from aldehydes, and, instead of two, there is only one hydrogen atom and one alkyl substituent bound to the (HOO)_2_C moiety. It is noteworthy that aldehyde-based unsupported di(hydroperoxy)alkanes are unstable oils that are hard to purify and handle because they decompose swiftly and form mixtures with the ensuing hydroxy(hydroperoxy)alkanes [12]. Therefore, another goal was to probe whether phosphine oxides could also stabilize the aldehyde-derived di(hydroperoxy)alkanes and render the adducts Cy_3_PO∙(HOO)_2_CHR crystalline and easy to handle.

In the following sections, it is demonstrated that in spite of reducing the size and weight of the peroxides substantially, the new di(hydroperoxy)alkane adducts of tricyclohexylphosphine oxide can easily be synthesized, crystallized, and characterized. Their interesting structural features are discussed along with their NMR and IR characteristics, and their superb solubility in common organic solvents is quantified.

## 2. Results and Discussion

### 2.1. Synthesis and Characterization

**Figure 1:**
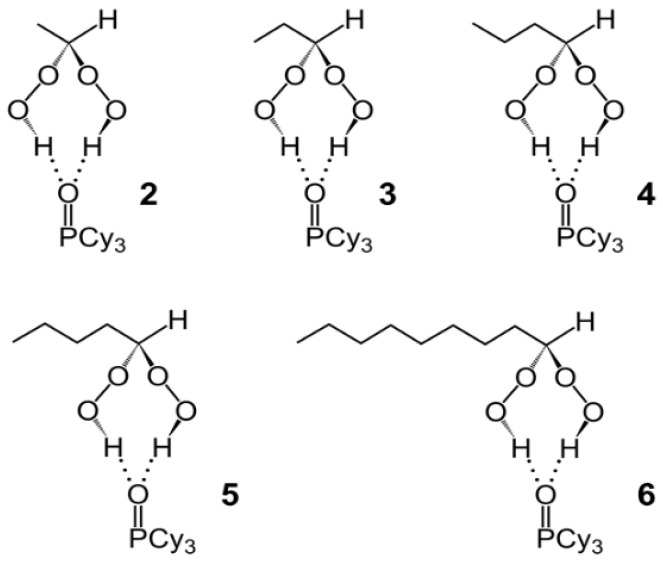
Di(hydroperoxy)alkane adducts **2**–**6** of Cy_3_PO (**1**).

The syntheses of the di(hydroperoxy)alkane adducts of tricyclohexylphosphine oxide **2**–**6** (Figure 1) were straightforward and followed one standard procedure. Combining DCM solutions of the clean phosphine oxide **1** [22] with the di(hydroperoxy)alkanes [12] in a 1:1 ratio, the adducts **2**–**6** formed instantly and could be obtained in crystalline form by the slow evaporation of the solvent at ambient temperature under the atmosphere. All adducts **2**–**6** have been obtained with a stoichiometrically precise 1:1 composition of peroxide and phosphine oxide.

The colorless adducts **2**–**6** (Figure 1) are stable at ambient temperature and only start to decompose during melting at temperatures higher than 84 °C. Besides the melting characteristics, the single crystal X-ray structures [48], the IR spectra [49], and the ^1^H, ^13^C, and ^31^P NMR spectroscopic data are reported for the adducts. Furthermore, selected solubilities in representative organic solvents have been quantified.

### 2.2. X-Ray Crystallography of the Adducts Cy_3_PO·(HOO)_2_CHR

**Figure 2:**
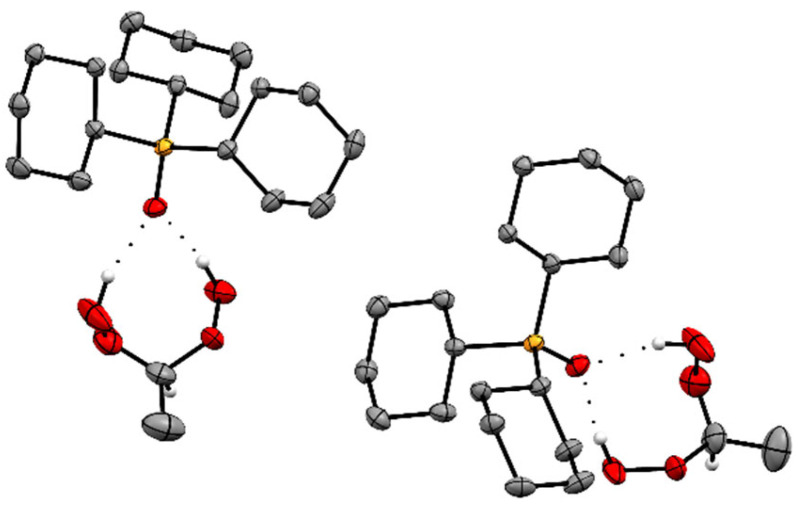
Two adduct assemblies of Cy_3_PO·(HOO)_2_CHCH_3_ (**2**). Hydrogen atoms except those in the CH(OOH)_2_ moieties are omitted for clarity.

**Figure 3:**
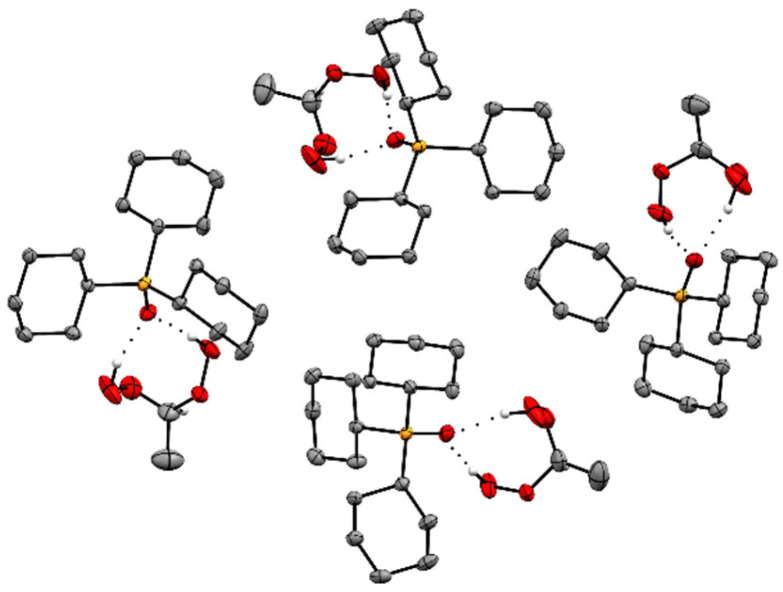
Stacking pattern of four adduct assemblies of Cy_3_PO·(HOO)_2_CHCH_3_ (**2**). Hydrogen atoms except those in the CH(OOH)_2_ moieties are omitted for clarity.

**Figure 4:**
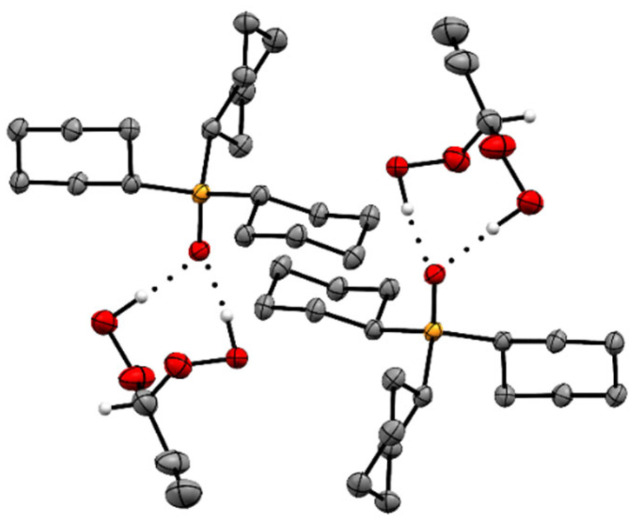
Stacking pattern of two adduct assemblies of Cy_3_PO·(HOO)_2_CHCH_2_CH_3_ (**3**). Hydrogen atoms except those in CH(OOH)_2_ moieties are omitted for clarity.

**Figure 5:**
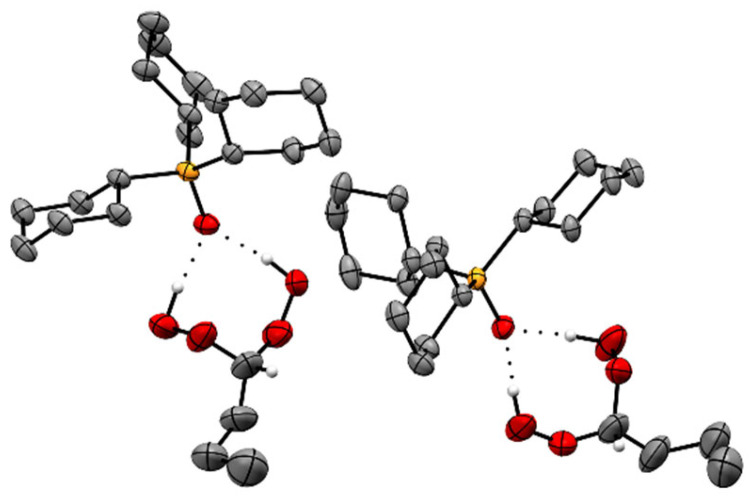
Stacking pattern of two adduct assemblies of Cy_3_PO·(HOO)_2_CH(CH_2_)_2_CH_3_ (**4**). Hydrogen atoms except those in CH(OOH)_2_ moieties are omitted for clarity.

**Figure 6:**
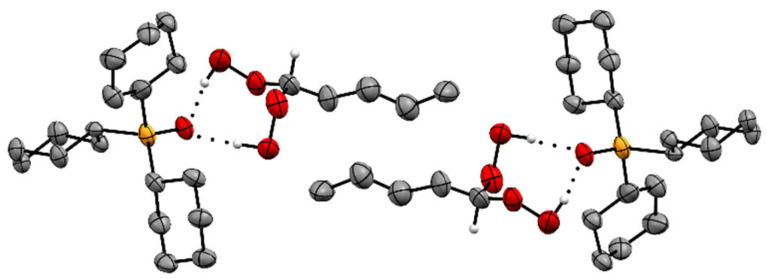
Stacking pattern of two adduct assemblies of Cy_3_PO·(HOO)_2_CH(CH_2_)_3_CH_3_ (**5**). Hydrogen atoms except those in CH(OOH)_2_ moieties are omitted for clarity.

**Figure 7:**
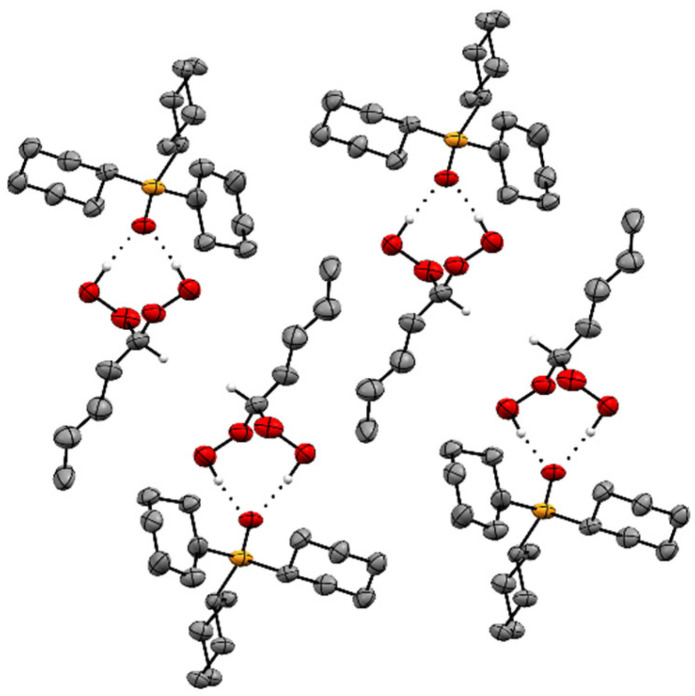
Stacking pattern of four adduct assemblies of Cy_3_PO·(HOO)_2_CH(CH_2_)_3_CH_3_ (**5**). Hydrogen atoms except those in CH(OOH)_2_ moieties are omitted for clarity.

**Figure 8:**
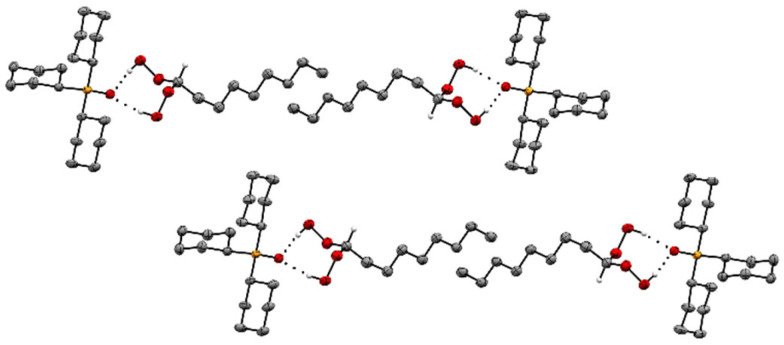
Stacking pattern of two adduct assemblies of Cy_3_PO·(HOO)_2_CH(CH_2_)_7_CH_3_ (**6**). Hydrogen atoms except those in CH(OOH)_2_ moieties are omitted for clarity.

The adducts Cy_3_PO·(HOO)_2_CHR **2**–**6** (Figure 1) crystallize readily in large colorless specimens of single crystal X-ray quality. The ease of crystallization is most probably due to the Cy_3_PO carrier that is favorable for packing the adducts in crystal lattices. More recently, we reported that various R_3_POs also enable the crystallization of hydrogen-bonded H_2_O_2_ and ketone-derived di(hydroperoxy)alkanes [21,22,23,24,25,26,27,28,29]. The single crystal X-ray structures of the adduct assemblies Cy_3_PO·(HOO)_2_CHR (**2**–**6**) are displayed in Figure 2 and Figure 3 (**2**), Figure 4 and Figure S1 (**3**), Figure 5 and Figure S2 (**4**), Figure 6 and Figure 7 (**5**), and Figure 8 (**6**) [48]. Relevant structural data are summarized in Table 1 and Table 2 and Tables S1 and S2 in the Supplementary Materials. The single crystal X-ray structures of all adducts **2**–**6** confirm the stoichiometrically precise 1:1 composition of the phosphine oxide and the di(hydroperoxy)alkane.

Interestingly, crystalline **2** (Figure 2) does not follow the arrangement of adduct assemblies observed with all adducts of the type R_3_PO·(HOO)_2_CR′R″ (R, R′, R″ = alkyl, aryl) [21,22,23,24,25,26,27,28,29]. The latter adducts, without exception, feature two adducts with the P=O groups being aligned and pointing in opposite directions. This conventional arrangement is observed also for **3** (Figure 4 and Figure S1). In **2**, the P=O groups are nearly perpendicular, and the overall stacking pattern is unprecedented and resembles a molecular roundabout (Figure 3). We assume that the unusual stacking of **2** is due to the small steric demand of the di(hydroperoxy)ethane moiety that is unable to fill the void created by the large Cy_3_PO of the adjacent assembly.

The larger propyl groups in the assemblies of adduct **3**, on the other hand, allow for the conventional, antiparallel stacking with the P=O groups pointing in opposite directions (Figure 4 and Figure S1). The minor space requirement of the hydrogen atom in the (HOO)_2_CH group as compared to adducts with all alkyl substituents described previously [21,22,23,24,25,26,27,28,29] does not impact the structure of **3,** and the usual stacking motif is realized.

Differing from the scenario of **2**, **3**, and structures reported previously [21,22,23,24,25,26,27,28,29], the adduct assemblies of **4** show another different packing motif (Figure 5 and Figure S2). In this case, the P=O groups of two adjacent adducts are nearly parallel and oriented in the same direction. This unprecedented packing motif produces a crowded scenario in the stacking pattern (Figure S2) and a noticeable kink at the terminal end of the butyl group.

The adduct assemblies of **5** follow the usual stacking motif with the P=O groups being aligned and pointing in opposite directions (Figure 6 and Figure 7). The pentyl chains perfectly align parallel to each other and create a well-organized pattern with optimal space filling. The adduct assemblies of **6** also align in the classic manner of two assemblies each oriented antiparallel to each other (Figure 8). Regarding **6**, it is remarkable that the methylene chains are fully extended. A similar scenario has been described for diphosphine dioxides with long alkyl chains previously [38]. Interestingly, the long methylene chains are not aligned over their full length like in **5** but only over the three terminal carbon atoms. Overall, the structure of **6** resembles a tenside with hydrophobic and hydrophilic sections.

The P=O bond lengths in **2**–**6** (Table 1) are in the same range as those observed for the doubly hydrogen-bonded peroxide adducts of diverse trialkyl- and triarylphosphine oxides of the types (R_3_PO·H_2_O_2_)_2_ (bond length range 1.4882 to 1.5046 Å) [18,19,20] and R_3_PO·(HOO)_2_CR′R″ (1.4992 to 1.5047 Å) [24,25,26,27,28,29]. Due to the strong hydrogen bonding of the di(hydroperoxy)alkanes to the oxygen atom in the P=O bonds, the latter are elongated in the adducts **2**–**6** as compared to the neat phosphine oxide **1** (Table 1). The bond length differences ∆(P=O) range between 0.0169 Å and 0.0253 Å.

Therewith, the P=O bonds in **2**–**6** are lengthened to about the same extent as observed for ketone-derived adducts [24,25,26,27,28,29]. The weakening of the P=O bonds due to adduct formation is confirmed by ^31^P NMR and IR spectroscopy (see below). The differences in the ∆(P=O) values of the diverse adducts **2**–**6** do not follow a trend (Table 1). Therefore, it can be assumed that the methylene chain length does not play a role, and the slight differences in the ∆(P=O) values between the adducts **2**–**6** are due to crystal packing effects.

Another criterion for the strength of the hydrogen bonds in the adducts **2**–**6** is the distance between the oxygen atoms in the O–H···O bridges. Distances between 2.75 and 2.85 Å are regarded as typical for these oxygen–oxygen distances [50,51]. Nearly all of the distances found for the adducts **2**–**6** are even shorter, with values ranging from 2.689 Å to 2.751 Å (Table 1). Therefore, it can be concluded that the phosphine oxide forms two strong hydrogen bonds with the di(hydroperoxy)alkanes. Again, there is no obvious trend that would link the oxygen–oxygen distance in the O–H···O assembly with the length of the alkyl chains.

Finally, we considered the bond angles at the CH carbon atoms of the di(hydroperoxy)alkane moieties in **2**–**6** (Table 2). These angles should indicate whether the formation of the two nearly linear hydrogen bonds to the phosphine oxide leads to a distortion of the tetrahedral geometry in order to accommodate the packing in the single crystals. All O–C–O angles of **2**–**6** fall within the narrow range of 112.5° and 114.5° and, therewith, are substantially larger than the tetrahedral angle 109.5°. The values are even larger than those of comparable adducts incorporating cyclic alkanes [28]. For example, the O–C–O angle in the adduct Cy_3_PO·(HOO)_2_C(CH_2_)_4_ where the quaternary carbon is part of a cyclopentyl ring amounts to only 111.8° [28]. Regarding the O–C–C angles of **2**–**6** (Table 2), the values scatter between 103.8° and 121.2°. Both extreme values are found in adduct **2**, which also shows a very different packing of the adduct assemblies in the unit cell (Figure 3). Overall, the bond angles around the di(hydroperoxy)alkane CH carbon in **2**–**6** reflect the accommodation of the individual structures to the packing motif of the adducts.

### 2.3. NMR Spectroscopy of the Adducts of Cy_3_PO·(HOO)_2_CHR

All NMR spectra of **2**–**6** are displayed in the Supplementary Materials (Figures S3–S13). ^1^H and ^13^C NMR spectroscopies prove the successful transformation of the starting aldehydes into the di(hydroperoxy)alkanes. The most diagnostic resonance stems from the aldehyde proton C*H*O that is located between 9.4 to 9.8 ppm for all alkyl aldehydes used in this study. This signal vanishes during the formation of the adducts **2**–**6,** and a new signal in the range from 5.01 ppm to 5.24 ppm appears, corresponding to the protons in the (HOO)_2_C*H* moieties (Figures S4–S8). The ^3^*J*(H-H) coupling to the adjacent CH_3_ protons for **2** and CH_2_ protons for **3**–**6** splits these signals into a characteristic quartet and triplets, respectively. In ^13^C NMR, the transitions from the aldehydes to the di(hydroperoxy)alkanes manifest in the *C*HO resonance of the alkyl aldehydes in the range from 200 ppm to 203 ppm [52] disappearing and new signals between 110.94 ppm and 106.26 ppm emerging for the (HOO)_2_*C*H moieties (Figures S9–S13). All ^13^C NMR signals could be assigned by comparison with different adducts described previously [22,23,24,25,26,27,28,29] and by using chemical shift tables (Figures S9–S13) [52]. Furthermore, the ^n^*J*(^31^P-^13^C) couplings that were only visible for the cyclohexyl carbon signals and were not propagated beyond the hydrogen bonds were utilized. Where needed, HMBC and HSQC spectra were recorded.

While ^1^H and ^13^C NMR spectroscopies clearly indicate the transformation of the aldehydes to the corresponding di(hydroperoxy)alkanes, the proof of adduct formation is based on ^31^P NMR. Compared to the chemical shift of Cy_3_PO (49.91 ppm), the ^31^P NMR signals of the adducts **2**–**6** are substantially downfield-shifted by more than 7 ppm (Table 3, Figure S3). The hydrogen bonding of the P=O group to the two hydroperoxy groups in the adducts reduces the electron density at the phosphorus nucleus. As a result, the signal of the deshielded ^31^P nucleus is shifted downfield. Similar changes in the ^31^P NMR chemical shifts have been described for other di(hydroperoxy)alkane adducts of phosphine oxides previously, with the largest chemical shift changes being observed for adducts of Cy_3_PO [28].

### 2.4. IR Spectroscopy of the Adducts of Cy_3_PO·(HOO)_2_CHR

The IR stretching frequencies [49,53] for the O–H and P=O groups of the adducts **2**–**6** are summarized in Table 4. The IR data complement the results from the single crystal X-ray diffraction and ^31^P NMR measurements. The strong hydrogen bonds in the adducts manifest in a weakening of the P=O bond and correspondingly lower wavenumbers in **2**–**6** as compared to the neat phosphine oxide **1**. This effect is rather pronounced, and the differences amount to 25 to 30 cm^−1^. These differences ∆*ν*(P=O) are in the same order of magnitude as those obtained for di(hydroperoxy)alkanes of the type R_3_PO·(HOO)_2_CR′R″ hydrogen-bonded to the electron-rich Cy_3_PO reported previously [24,25,26,27,28,29].

Another consequence of the adduct formation is that the O–H bond is weakened in **2**–**6** by the strong hydrogen bonds with the P=O group of the phosphine oxide carrier. In nonpolar solvents, dilute substances containing OH groups exhibit sharp absorption peaks between 3650 cm^−1^ and 3590 cm^−1^ [49]. However, the O–H stretching bands for **2**–**6** are found at much lower wavenumbers and within the narrow range from 3193 cm^−1^ to 3200 cm^−1^ (Table 4). This result confirms that in the solid adducts, the P=O groups are firmly hydrogen-bonded to the di(hydroperoxy)alkane moieties.

### 2.5. Solubilities of the Adducts in Organic Solvents

Aqueous H_2_O_2_ is a very potent oxidizer and used extensively in academia and industry [1,2,3]. However, besides its lack of shelf stability, high cost, and safety issues, the crucial drawback is that most oxidations have to be performed in biphasic mixtures. Since the actual reaction only occurs at the interface between the aqueous and organic solvent layers, longer reaction times are required. Furthermore, the work-up necessitates additional phase separation and product-drying steps. Water-sensitive educts or products are not amenable to treatment with aqueous H_2_O_2_. Recently, we succeeded in immobilizing a phosphine oxide carrier on a silica surface that stabilized H_2_O_2_ and di(hydroperoxy)alkanes by strong hydrogen bonds [21]. This material allowed the use of diverse protic and nonprotic solvents in oxidation reactions because the peroxide was exposed to the substrate in a monolayer on the mesoporous high-surface area silica [21]. However, this method requires an immobilization step and support material.

**Figure 9:**
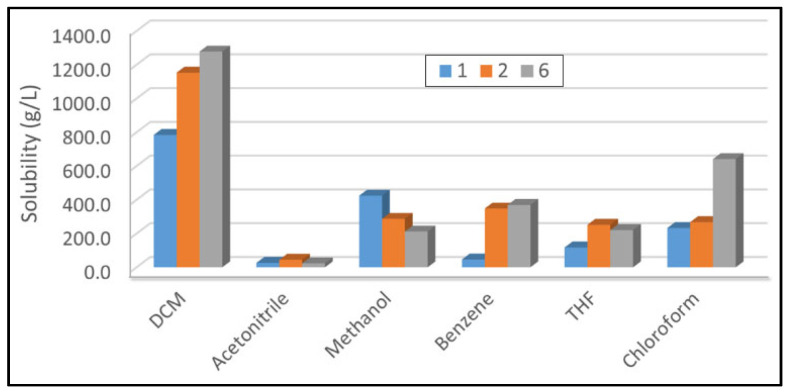
Solubilities of the adducts Cy_3_PO·(HOO)_2_CHCH_3_ (**2**) and Cy_3_PO·(HOO)_2_CH(CH_2_)_7_CH_3_ (**6**) in representative organic solvents, compared to Cy_3_PO (**1**).

Earlier, favorable phosphine oxides have been utilized to render H_2_O_2_ and di(hydroperoxy)alkanes soluble in organic solvents [22,23,24,25,26,27,28,29]. The phosphine oxide Cy_3_PO should be uniquely suited for increasing the solubilities of the peroxide moieties in the adducts **2**–**6**. In fact, the quantified solubilities of the adducts that incorporate the shortest and longest alkyl chains, Cy_3_PO·(HOO)_2_CHCH_3_ (**2**) and Cy_3_PO·(HOO)_2_CH(CH_2_)_7_CH_3_ (**6**), in representative organic solvents are very high (Figure 9). In most solvents, the adducts **2** and **6** are even more soluble than the carrier Cy_3_PO (**1**). The only exception is the protic solvent methanol, which might lead to the dissociation of the adduct hydrogen bonds in solution. The higher solubilities of the smaller fragments of **2,** as compared to those of **6** with the hydrophobic octyl substituent at the (HOO)_2_CH group, support the assumption of dissociation in methanol. The solubilities of **2** and **6** are remarkably high in the aromatic solvent benzene and the chlorinated solvents DCM and chloroform. In DCM, for example, more than 1.2 g of **6** are soluble per mL of DCM, and nearly 1.1 g per mL of **2** can be dissolved (Figure 9). The polar, non-protic solvent acetonitrile is the least favorable solvent for all species, **1**, **2**, and **6**.

In summary, the adducts of Cy_3_PO·(HOO)_2_CHR are highly soluble in many organic solvents without the decomposition of the peroxide moiety. Therefore, in contrast to aqueous hydrogen peroxide or insoluble inorganic peroxides, they can be applied for oxidation reactions in one homogeneous organic phase. The solubilities of the new adducts in non-protic and chlorinated solvents allow a broader range of applications for oxidation reactions. Biphasic liquid/liquid and liquid/solid reaction mixtures that prolong the reaction times, complicate the work-up, and diminish the product yields and selectivities can be avoided by choosing any of the adducts **2**–**6**.

### 2.6. Application for the Selective Oxidation of PPh_3_

Finally, we sought to demonstrate that the new adducts of the type Cy_3_PO·(HOO)_2_CHR (R = alkyl) are able to selectively oxidize phosphines in analogy to the ketone-derived adducts described previously [21,22,23,24,25,26,27,28,29]. We chose adduct **6** in combination with PPh_3_ as the substrate because triarylphosphines are not oxidized in air, even in solution and at high temperatures [22]. The outcome of the oxidation reaction can be determined by ^31^P NMR spectroscopy. The original resonance at about –6 ppm vanishes, while the signal for OPPh_3_ appears at 29.10 ppm [22]. Another question about the new adducts can be answered unequivocally with this experiment. After the active oxygen atoms of the adduct are spent, the aldehyde is obtained again. This is clearly visible in the ^1^H and ^13^C NMR spectra (Figures S14 and S15). The aldehyde proton signal at about 9.5 ppm reappears in the ^1^H NMR spectrum after the reaction, matching the corresponding peak in the spectrum of nonanal. Analogously, the ^13^C NMR spectrum of the reaction mixture displays the aldehyde carbon signal at about 203 ppm, while the resonance of the adduct **6** that stems from the carbon atom attached to the two hydroperoxy groups at ca. 110 ppm has vanished. In summary, this reaction shows that the new adduct is a powerful oxidant that led to the full and selective oxidation of a triarylphosphine within minutes and that the reaction product besides the oxidized species and the supporting phosphine oxide Cy_3_PO is the corresponding aldehyde.

## 3. Experimental Section

General Considerations**.** All reactions were carried out under the atmosphere unless mentioned otherwise. Cy_3_PO was synthesized water-free from the phosphine using air oxygen after adsorption on activated carbon (AC) as described earlier [54]. The absence of water was checked by IR spectroscopy. The solvents, hydrogen peroxide (30% aqueous solution), and aldehydes were used as obtained from the supplier. All di(hydroperoxy)alkanes and their Cy_3_PO adducts **2**–**6** were synthesized in analogy to the representative procedure outlined for **2** below. The ^31^P (Figure S3), ^1^H (Figures S4–S8), and ^13^C NMR spectra (Figures S9–S13) were recorded at ambient temperature on a Bruker 400 MHz NMR instrument at 161.82, 399.76, and 100.53 MHz, respectively. The ^31^P and ^13^C NMR spectra were proton-decoupled. For referencing the ^31^P NMR spectra, neat Ph_2_PCl (*δ*(^31^P) = +81.92 ppm), placed in a capillary that was centered in the 5 mm NMR tube, was used. The ^1^H and ^13^C NMR spectra were referenced using the signals of the solvent CDCl_3_ (residual protons: *δ*(^1^H) = 7.26 ppm; *δ*(^13^C) = 77.16 ppm). The IR spectra of the neat powders of the adducts **2**–**6** were obtained with a Shimadzu IRAffinity-1 FTIR spectrometer equipped with a Pike Technologies MIRacle ATR plate.

Representative Synthesis of Cy_3_PO∙(HOO)_2_CHCH_3_ (2). Syntheses of (HOO)_2_CHCH_3_: acetaldehyde (120.0 mg, 2.727 mmol, 1.000 eq.), phosphomolybdic acid (83.3 mg, 0.0456 mmol, 0.0167 eq.), and MgSO_4_ (448.7 mg, 3.728 mmol, 1.367 eq.) were added to 14 mL of 3-fold concentrated ethereal H_2_O_2_ solution [12] in a reaction vial and stirred for 24 h at ambient temperature. The solids were filtered off through Celite and washed with 10 mL of EtOAc. EtOAc (10 mL) and H_2_O (10 mL) were added to the combined filtrate and washing solutions. Then, the organic and aqueous phases were separated with a separatory funnel. The aqueous layer was extracted two times with 20 mL portions of EtOAc, and all organic phases were combined. They were washed with 15 mL of H_2_O and 15 mL of a brine solution. Finally, the organic phase was dried over anhydrous Na_2_SO_4_. The Na_2_SO_4_ was filtered off, and the organic phase was stripped of the solvent by rotary evaporation. The resulting oily residue was subjected to oil pump vacuum, and the di(hydroperoxy)ethane was obtained as slightly yellow oil (196.8 mg, 2.094 mmol, and 76.77% yield with respect to acetaldehyde). All other di(hydroperoxy)alkanes were synthesized according to this representative procedure and were obtained as colorless oils with 52–64% yields with respect to their corresponding aldehydes.

Adduct synthesis: tricyclohexylphosphine oxide (**1**, 96.8 mg, 0.327 mmol, 1.00 eq.) and di(hydroperoxy)ethane (39.4 mg, 0.419 mmol, 1.28 eq.) were dissolved in 1 mL of DCM, and the reaction mixture was homogenized by treatment with a Pasteur pipette for 1 min. The solution was placed on a watch glass, and the solvent was allowed to evaporate overnight under ambient conditions. The resulting solid was scraped off and dried further under vacuum. The adduct Cy_3_PO∙(HOO)_2_CHCH_3_ (**2**) was obtained as a white powder (109.8 mg, 0.2812 mmol, 86.1% isolated yield with respect to **1**). All of the other adducts (**3**–**6**) were synthesized following the same procedure, except for one additional step. After drying **3**–**6** overnight on a watch glass, the adducts were washed five times with 1 mL portions of hexanes to obtain the pure products. After further drying under ambient conditions overnight, the adducts **3**–**6** were obtained as white powders with 29–54% isolated, not-optimized yields with respect to **1**. For obtaining single crystals of X-ray quality, see Supplementary Materials.

NMR data of **2** (*δ*, CDCl_3_). ^31^P{^1^H} 57.35 (s); ^1^H 11.98 (O*H*), 5.24 (q, ^3^*J(*^1^H–^1^H) = 5.6 Hz, 1H, OC*H*), 1.87 (br d, 6H, ^2^*J*(^1^H–^1^H) = 11.3 Hz, PCHC*H*eq), 1.84–1.75 (m, 9H, PC*H*_ax_CH_2_C*H*_eq_), 1.75–1.61 (m, 3H, PCH(CH_2_)_2_C*H*_eq_), 1.50–1.30 (m, 6H, PCHC*H*_ax_), 1.33 (d, ^3^*J(*^1^H–^1^H) = 5.6 Hz, 3H, C*H*_3_), 1.30–1.12 (m, 9H, PCHCH_2_C*H*_ax_C*H*_ax_). ^13^C{^1^H} 106.26 (s, O*C*), 34.89 (d, ^1^*J*(^31^P–^13^C) = 60.6 Hz, P*C*), 26.81 (d, ^3^*J*(^31^P–^13^C) = 11.9 Hz, PC_2_*C*), 26.08 (d, ^2^*J*(^31^P–^13^C) = 2.8 Hz, PC*C*), 26.01 (s, PC_3_*C*), 15.10 (s, *C*H_3_).

NMR data of **3**. (*δ*, CDCl_3_). ^31^P{^1^H} 57.05 (s); ^1^H 11.99 (O*H*), 5.01 (t, ^3^*J*(^1^H-^1^H) = 5.8 Hz, 1H, OC*H*), 1.90 (br d, 6H, ^2^*J*(^1^H–^1^H) = 11.0 Hz, PCHC*H*_eq_), 1.86–1.78 (m, 9H, PC*H*_ax_CH_2_C*H*_eq_), 1.78–1.63 (m, 3H, PCH(CH_2_)_2_C*H*_eq_), 1.72 (quint, ^3^*J(*^1^H–^1^H) = 5.8 Hz, 2H, OCHC*H*_2_), 1.53–1.33 (m, 6H, PCHC*H*_ax_), 1.33–1.13 (m, 9H, PCHCH_2_C*H*_ax_C*H*_ax_), 0.99 (t, ^3^*J(*^1^H–^1^H) = 7.4 Hz, 3H, C*H*_3_). ^13^C{^1^H} 110.94 (s, O*C*), 34.91 (d, ^1^*J*(^31^P–^13^C) = 60.8 Hz, P*C*), 26.86 (d, ^3^*J*(^31^P–^13^C) = 11.7 Hz, PC_2_*C*), 26.14 (s, PC*C*), 26.05 (s, PC_3_*C*), 22.29 (s, OC*C*), 9.40 (s, *C*H_3_).

NMR data of **4**. (*δ*, CDCl_3_). ^31^P{^1^H} 56.96 (s); ^1^H 11.96 (O*H*), 5.09 (t, ^3^*J(*^1^H–^1^H) = 6.0 Hz, 1H, OC*H*), 1.90 (dd, 6H, ^2^*J*(^1^H–^1^H) = 2.4 Hz, ^2^*J*(^1^H–^1^H) = 10.9 Hz, PCHC*H*_eq_), 1.87–1.77 (m, 9H, PC*H*_ax_CH_2_C*H*_eq_), 1.77–1.61 (m, 3H, PCH(CH_2_)_2_C*H*_eq_), 1.67 (dt, ^3^*J(*^1^H–^1^H) = 6.4 Hz, ^3^*J(*^1^H–^1^H) = 9.0 Hz, 2H, OCHC*H*_2_), 1.55–1.32 (m, 6H, PCHC*H*_ax_), 1.46 (sextet, ^3^*J(*^1^H–^1^H) = 7.7 Hz, 2H, OCHCH_2_C*H*_2_), 1.33–1.12 (m, 9H, PCHCH_2_C*H*_ax_C*H*_ax_), 0.92 (t, ^3^*J(*^1^H–^1^H) = 7.4 Hz, 3H, C*H*_3_); ^13^C{^1^H} 109.69 (s, O*C*), 34.88 (d, ^1^*J*(^31^P–^13^C) = 60.5 Hz, P*C*), 30.92 (s, OC*C*), 26.85 (d, ^3^*J*(^31^P–^13^C) =11.9 Hz, PC_2_*C*), 26.12 (d, ^2^*J*(^31^P–^13^C) = 2.9 Hz, PC*C*), 26.04 (s, PC_3_*C*), 18.35 (s, OC_2_*C*), 13.99 (s, *C*H_3_).

NMR data of **5**. (*δ*, CDCl_3_). ^31^P{^1^H} 57.20 (s); ^1^H 12.01 (O*H*), 5.08 (t, ^3^*J(*^1^H–^1^H) = 6.0 Hz, 1H, OC*H*), 1.90 (dd, 6H, ^2^*J*(^1^H–^1^H) = 2.5 Hz, ^2^*J*(^1^H–^1^H) = 11.1 Hz, PCHC*H*_eq_), 1.86–1.78 (m, 9H, PC*H*_ax_CH_2_C*H*_eq_), 1.78–1.63 (m, 3H, PCH(CH_2_)_2_C*H*_eq_), 1.70 (dt, ^3^*J(*^1^H–^1^H) = 6.5 Hz, ^3^*J(*^1^H–^1^H) = 9.0 Hz, 2H, OCHC*H*_2_), 1.56–1.32 (m, 6H, PCHC*H*_ax_), 1.56–1.39 (quintet, 2H, OCHCH_2_C*H*_2_), 1.33 (sextet, ^3^*J(*^1^H–^1^H) = 7.5 Hz, 2H, OCHCH_2_CH_2_C*H*_2_), 1.32–1.14 (m, 9H, PCHCH_2_C*H*_ax_C*H*_ax_), 0.89 (t, ^3^*J(*^1^H–^1^H) = 7.2 Hz, 3H, C*H*_3_); ^13^C{^1^H} 109.89 (s, O*C*), 34.99 (d, ^1^*J*(^31^P–^13^C) = 60.6 Hz, P*C*), 28.67 (s, OC*C*), 27.17 (s, OC_2_*C*), 26.90 (d, ^3^*J*(^31^P–^13^C) = 11.9 Hz, PC_2_*C*), 26.20 (d, ^2^*J*(^31^P–^13^C) = 2.9 Hz, PC*C*), 26.10 (s, PC_3_*C*), 22.60 (s, OC_3_*C*), 14.05 (s, *C*H_3_).

NMR data of **6**. (*δ*, CDCl_3_). ^31^P{^1^H} 57.43 (s); ^1^H 11.96 (O*H*), 5.07 (t, ^3^*J(*^1^H–^1^H) = 6.0 Hz, 1H, OC*H*), 1.89 (dd, 6H, ^2^*J*(^1^H–^1^H) = 2.6 Hz, ^2^*J*(^1^H–^1^H) = 10.9 Hz, PCHC*H*_eq_), 1.86–1.77 (m, 9H, PC*H*_ax_CH_2_C*H*_eq_), 1.77–1.61 (m, 3H, PCH(CH_2_)_2_C*H*_eq_), 1.68 (dt, ^3^*J(*^1^H–^1^H) = 6.2 Hz, ^3^*J(*^1^H–^1^H) = 9.3 Hz, 2H, OCHC*H*_2_), 1.56–1.33 (m, 6H, PCHC*H*_ax_), 1.56–1.15 (m, 12H, OCHCH_2_(C*H*_2_)_6_CH_3_), 1.33–1.15 (m, 9H, PCHCH_2_C*H*_ax_C*H*_ax_), 0.85 (t, ^3^*J(*^1^H–^1^H) = 7.0 Hz, 3H, C*H*_3_); ^13^C{^1^H} 109.91 (s, O*C*), 34.87 (d, ^1^*J*(^31^P–^13^C) = 60.6 Hz, P*C*), 31.96 (s, OC_6_*C*), 29.52 (s, OC_3_*C*)*, 29.49 (s, OC_5_*C*)*, 29.30 (s, OC_4_*C*)*, 28.95 (s, OC*C*), 26.85 (d, ^3^*J*(^31^P–^13^C) = 11.9 Hz, PC_2_*C*), 26.11 (d, ^2^*J*(^31^P–^13^C) = 2.8 Hz, PC*C*), 26.03 (s, PC_3_*C*), 25.01 (s, OC_2_*C*), 22.76 (s, OC_7_*C*), 14.20 (s, *C*H_3_). * Assignments interchangeable.

Melting ranges. The melting ranges of the adducts **2**–**6** (Table 5) were obtained using sealed capillaries and a conventional melting point apparatus (Optimelt). All melting ranges were lower than the melting point of neat Cy_3_PO (155–157 °C). The adducts **2**–**6** started melting at the given lower values and reached the clear points at the high values. The reason why the adducts have no single melting points is because they start to decompose at the temperatures required for melting.

Solubilities of **1**, **2**, and **6**. The corresponding phosphine oxide or adduct (10–16 mg) was weighed in a 20 mL vial. The selected solvent was added in dropsized portions to the vial while manually shaking it. The temperature was maintained at 20 °C. Once all solid was dissolved, the weight of the added solvent was recorded, and the solvent volume was calculated.

Oxidation of PPh_3_. Adduct **6** (22.0 mg, 0.0451 mmol, 1 equiv.) was added to PPh_3_ (20.0 mg, 0.0762 mmol, 1.69 equiv.), dissolved in 0.5 mL CDCl_3_. The reaction mixture was stirred for 5 min at RT and subsequently analyzed with ^1^H, ^13^C, and ^31^P NMR spectroscopies.

IR Spectroscopy. The IR spectra of the polycrystalline materials were recorded using a Shimadzu IRAffinity-1 FTIR spectrometer equipped with a Pike Technologies MIRacle ATR plate.

X-Ray Diffraction. See Supplementary Materials and references [55,56,57,58,59].

## 4. Conclusions

Five representative hydrogen-bonded peroxide adducts of the novel type Cy_3_PO·(HOO)_2_CHR (R = alkyl) (**2**–**6**) have been synthesized and fully characterized. Single crystal X-ray diffraction studies confirmed that all adducts feature a precise 1:1 composition of di(hydroperoxy)alkane moiety and the hydrogen-bonded phosphine oxide. The arrangements of the adduct assemblies in the solid state follow the classic pattern for **3**, **5**, and **6**, but different new packing motifs are found for **2** and **4**. In accordance with the elongation of the P=O bonds detected by X-ray diffraction, the ^31^P NMR spectra display downfield-shifted signals, and the IR wavenumbers ν(P=O) of all adducts are smaller as compared to the values of neat Cy_3_PO. The solubilities of the adducts are high in common organic solvents and have been quantified for **1,** for comparison, and **2** and **6**.

In summary, the described new adducts, derived from aldehydes, represent a hitherto missing link of di(hydroperoxy)alkane adducts of phosphine oxides. It has been demonstrated that this new type of adduct is stable, crystallizes easily, and is amenable to full spectroscopic characterization. The strong hydrogen bonds between the peroxide and phosphine oxide result in shelf-stable, crystalline material with high solubility in organic solvents, allowing for oxidation reactions in one phase. As one preliminary application, a phosphine has been oxidized selectively to the phosphine oxide. The easy synthesis, handling, and dosing of these solid peroxides will make a positive impact on synthetic chemistry.

## Figures and Tables

**Table 1:** Distances O–H···O and P=O bond lengths in **2**–**6** (Å) and differences ∆(P=O) between the P=O bond lengths of the adducts Cy_3_PO·(HOO)_2_CHR (**2**–**6**) and the corresponding phosphine oxide Cy_3_PO (**1**) (1.490(2) Å).

Adduct	O–H···O (Å)	P=O (Å)	∆(P=O) (Å)
**2**	2.689/2.751	1.5071 (17)	0.0169 (17)
**3**	2.719/2.741	1.5110 (9)	0.0208 (9)
**4**	2.711/2.719	1.5082 (14)	0.0180 (14)
**5**	2.701/2.702	1.5074 (11)	0.0172 (11)
**6**	2.712/2.737	1.5155 (10)	0.0253 (10)

**Table 2:** Bond angles at the CH carbon atom of the di(hydroperoxy)alkane moieties in the adducts **2**–**6**.

Cy_3_PO·(HOO)_2_CHR	O–C–O (°)	O1–C–C/O2–C–C (°)
**2**	114.5 (2)	103.8 (3)/121.2 (3)
**3**	112.5 (3)	113.7 (3)/106.5 (3)
**4**	113.6 (3)	114.3 (5)/119.0 (5)
**5**	113.66 (17)	114.0 (2)/114.44 (19)
**6**	113.05 (12)	105.40 (12)/115.43 (12)

**Table 3:** ^31^P NMR chemical shifts. The chemical shift differences ∆*δ* (ppm) refer to the chemical shift of the phosphine oxide Cy_3_PO (49.91 ppm). The solvent was CDCl_3_ for all samples.

Cy_3_PO·(HOO)_2_CHR	*δ*(^31^P) (ppm)	∆*δ*(^31^P) (ppm)
**2**	57.35	7.44
**3**	57.05	7.14
**4**	56.96	7.05
**5**	57.20	7.29
**6**	57.43	7.52

**Table 4:** IR wavenumbers for the stretching vibrations of the O–H and P=O groups (cm^−1^) of the adducts **2**–**6**. The wavenumber differences ∆*ν*(P=O) (cm^−1^) refer to the ν(P=O) of the phosphine oxide Cy_3_PO (1157 cm^−1^) [22].

Cy_3_PO·(HOO)_2_CHR	ν(O–H) (cm^−1^)	ν(P=O) (cm^−1^)	∆ν(P=O) (cm^−1^)
**2**	3195	1129	28
**3**	3193	1127	30
**4**	3198	1131	26
**5**	3200	1131	26
**6**	3196	1132	25

**Table 5:** Melting ranges of the adducts **2**-**6**.

Cy_3_PO·(HOO)_2_CHR	mp (°C)
**2**	87–99
**3**	84–90
**4**	100–105
**5**	84–90
**6**	89–92

## Data Availability

CCDC 2404335 (**2**), 2404336 (**3**), 2404337 (**4**), 2404338 (**5**), and 2404339 (**6**) contain the crystallographic data for this paper. These data can be obtained free of charge via www.ccdc.cam.ac.uk/data_request/cif (accessed on 19 December 2024), or by emailing data_request@ccdc.cam.ac.uk, or by contacting the Cambridge Crystallographic Data Centre, 12 Union Road, Cambridge CB2 1EZ, UK; fax: +44-1223-336033.

## Supplementary Materials

The following supporting information for X-ray diffraction [55,56,57,58,59] and NMR spectroscopy can be downloaded at https://www.mdpi.com/article/10.3390/molecules30020329/s1. Table S1: Crystallographic data for **2**, **3**, and **4**; Table S2: Crystallographic data for **5** and **6**; Figure S1: Stacking pattern of two adduct assemblies of Cy_3_PO·(HOO)_2_CHCH_2_CH_3_ (**3**). Hydrogen atoms except those in CH(OOH)_2_ moieties are omitted for clarity; Figure S2: Stacking pattern of four adduct assemblies of Cy_3_PO·(HOO)_2_CH(CH_2_)_2_CH_3_ (**4**). Hydrogen atoms except those in CH(OOH)_2_ moieties are omitted for clarity; Figure S3: ^31^P NMR spectra of the adducts **2**–**6** in CDCl_3_. All signals are downfield-shifted as compared to the resonance of Cy_3_PO (*δ*(^31^P) = 49.91 ppm); Figure S4: ^1^H NMR spectrum of the adduct Cy_3_PO·(HOO)_2_CHCH_3_ (**2**); Figure S5: ^1^H NMR spectrum of the adduct Cy_3_PO·(HOO)_2_CHCH_2_CH_3_ (**3**); Figure S6: ^1^H NMR spectrum of the adduct Cy_3_PO·(HOO)_2_CH(CH_2_)_2_CH_3_ (**4**); Figure S7: ^1^H NMR spectrum of the adduct Cy_3_PO·(HOO)_2_CH(CH_2_)_3_CH_3_ (**5**); Figure S8: ^1^H NMR spectrum of the adduct Cy_3_PO·(HOO)_2_CH(CH_2_)_7_CH_3_ (**6**); Figure S9: ^13^C NMR spectrum of the adduct Cy_3_PO·(HOO)_2_CHCH_3_ (**2**); Figure S10: ^13^C NMR spectrum of the adduct Cy_3_PO·(HOO)_2_CHCH_2_CH_3_ (**3**); Figure S11: ^13^C NMR spectrum of the adduct Cy_3_PO·(HOO)_2_CH(CH_2_)_2_CH_3_ (**4**); Figure S12: ^13^C NMR spectrum of the adduct Cy_3_PO·(HOO)_2_CH(CH_2_)_3_CH_3_ (**5**); Figure S13: ^13^C NMR spectrum of the adduct Cy_3_PO·(HOO)_2_CH(CH_2_)_7_CH_3_ (**6**). Assignments for C4, C5, and C6 are interchangeable; Figure S14: ^1^H NMR spectra of nonanal (top) and the reaction mixture after combining adduct **6** with 2 equivalents of PPh_3_ (bottom); Figure S15: ^13^C NMR spectra of nonanal (top), the adduct Cy_3_PO·(HOO)_2_CH(CH_2_)_7_CH_3_ (**6**) (middle), and the reaction mixture after combining adduct **6** with 2 equivalents of PPh_3_ (bottom). The supporting information is available free of charge. Detailed description of materials and methods used for X-ray crystallography. Crystallographic information for **2**–**5** with selected data in tables. ^31^P, ^13^C, and ^1^H NMR spectra.
