# Supplementary material for: Hydrogen-Bonded Di(hydroperoxy)alkane Adducts of the Type Cy3P=O·(HOO)2CHR (R = Alkyl)
Source: Molecules. 2025 Jan 15;30(2):329. doi: 10.3390/molecules30020329 (PMC11767494; doi:10.3390/molecules30020329)
Supplement: Supplementary file 1 [file molecules-30-00329-s001.zip › molecules-3410657-supplementary.pdf]

molecules

MDPI

Electronic Supplementary Material (ESI)

Hydrogen-Bonded Di(hydroperoxy)alkane Adducts of the Type $Cy_3P{=}O{\cdot}(HOO)_2CHR$ (R = Alkyl)

Rahym Ashirov, Maya Todorovic, Nattamai Bhuvanesh and Janet Blümel *

Department of Chemistry, Texas A&M University, College Station, TX 77842-3012, USA
* Correspondence: bluemel@tamu.edu

I. X-Ray Crystallography

1. A solution of **2** in 1:5 dichloromethane-hexanes was concentrated by slow evaporation. A colorless block with very well-defined faces from a representative sample of crystals of the same habit was collected and data were obtained as outlined in Table **S1.** The crystal was kept in a nitrogen stream at T = 100.0(4) K during measurement. Crystal screening, unit cell determination, and data collection were carried out using a XtaLAB Synergy, Dualflex, HyPix diffractometer. The diffraction patterns were indexed and the total number of runs and images were based on strategy calculations from the program CrysAlisPro [55]. Data were measured using ω scans with Cu K_α radiation. Absorption corrections were applied using CrysAlisPro [55]. The unit cells were refined using CrysAlisPro [55]. The structure was solved using ShelXT 2018/2 [56] and refined by full matrix least squares minimization on $\mathbf{F}^2$ using version 2019/1 of ShelXL 2019/1 [56]. All non-hydrogen atoms were refined anisotropically. Hydrogen atom positions were calculated geometrically and refined using the riding model. Olex2 and Mercury were employed for the final data presentation and for structure plots [57,58].

The crystal quality of **2** was not optimal. Most of the crystals were multiply twinned tending towards polycrystalline. A larger specimen of a crystal was cut to appropriate size to obtain a reasonable crystal to collect data. Elongated ellipsoids and residual electron density peaks near [O2,O3] and [O4,O5] suggested disorders which were successfully modeled between two positions each with an occupancy ratio of 0.78. Appropriate restraints were added to keep the bond distances, angles, and thermal ellipsoids of the disordered groups meaningful. Larger thermal ellipsoids on [O2A,O3A] suggested additional disorder. However, our trials to model the latter did not improve the refinement to any significance. Hydrogen atoms on O3 and O4 were located from the residual electron density peaks. H3AA, and H5AA were placed only to satisfy stoichiometry. The O-H distances were idealized. Remaining hydrogen atoms on the carbon atoms were placed geometrically using Olex2 [57].

2. A solution of **3** in dichloromethane was concentrated by slow evaporation. A colorless plate with very well-defined faces from a representative sample of crystals of the same habit was collected and data were obtained as outlined in Table **S2.** The crystal was kept in a nitrogen stream at T = 100.0(4) K during measurement. Crystal screening, unit cell determination, and data collection were carried out using a XtaLAB Synergy, Dualflex, HyPix

diffractometer. The diffraction patterns were indexed and the total number of runs and images were based on strategy calculations from the program CrysAlisPro [55]. The data were measured using ω scans with Cu K_α radiation. Absorption corrections were applied using CrysAlisPro [55] and the unit cells were refined using the same Program System [55]. The structure was solved using ShelXT 2018/2 [56]. The structure was refined by full matrix least squares minimization on $\mathbf{F}^2$ using version 2019/1 of ShelXL 2019/1 [56]. All non-hydrogen atoms were refined anisotropically. Most hydrogen atom positions were calculated geometrically and refined using the riding model, but some hydrogen atoms were refined freely. Olex2 and Mercury were employed for the final data presentation and structure plots [57,58].

Elongated ellipsoids and residual electron density near the peroxide moiety suggested disorder, which was successfully modeled between two positions with an occupancy ratio of 0.51. Appropriate restraints were used to keep the bond distances, angles, and thermal ellipsoids meaningful.

3. A solution of **4** in 1:1 carbon tetrachloride-toluene was concentrated by slow evaporation. A colorless block with very well-defined faces from a representative sample of crystals of the same habit was collected and data were obtained as outlined in Table **S1.** The crystal was kept in a nitrogen stream at T = 100.03(10) K during measurement. Crystal screening, unit cell determination, and data collection were carried out using a XtaLAB Synergy, Dualflex, HyPix diffractometer. The diffraction patterns were indexed and the total number of runs and images were based on strategy calculations from the program CrysAlisPro System [55]. Data were measured using ω scans with Cu K_α radiation. Absorption corrections were applied using CrysAlisPro [55] and the unit cells were refined using the same program System [55]. The structure was solved using ShelXT 2018/2 [56]. The structure was refined by full matrix least squares minimization on $\mathbf{F}^2$ using version 2019/1 of ShelXL 2019/1 [56]. All non-hydrogen atoms were refined anisotropically. Most hydrogen atom positions were calculated geometrically and refined using the riding model, but some hydrogen atoms were refined freely. Olex2 and Mercury were employed for the final data presentation and structure plots [57,58].

The crystal quality of **4** was not optimal. Several crystals were tried and the best data among them was used for analysis. Elongated thermal ellipsoids and residual electron density peaks near the peroxide moiety suggested disorder which was modeled between two positions with an occupancy ratio of 0.58. Appropriate restraints were added to keep the bond distances angles and thermal ellipsoids meaningful. Residual electron density peaks near the disorder group suggested further disorder. No efforts were made to model the latter. H3 and H5 were located from residual electron density peaks and were set riding on the respective oxygen atoms. All the remaining hydrogen atoms were placed geometrically and set riding on the respective parent atoms.

4. A solution of **5** in 1:1 dichloromethane-hexanes was concentrated by slow evaporation. A colorless plate with very well-defined faces from a representative sample of crystals of the same habit was collected and data were obtained as outlined in Table **S2.** The crystal was kept in a nitrogen stream at T = 100.00(10) K during measurement. Crystal screening, unit cell determination, and data collection were carried out using a XtaLAB Synergy, Dualflex, HyPix diffractometer. The diffraction patterns were indexed and the total number of runs and

images were based on strategy calculations from the program CrysAlisPro System [55]. Data were measured using ω scans with Cu K_α radiation. Absorption corrections were applied using CrysAlisPro [55] and the unit cells were refined using CrysAlisPro [55]. The structure was solved using ShelXT 2018/2 [56]. The structure was refined by full matrix least squares minimization on $\mathbf{F}^2$ using version 2019/1 of ShelXL 2019/1 [56]. All non-hydrogen atoms were refined anisotropically. Hydrogen atom positions were calculated geometrically and refined using the riding model. Olex2 and Mercury were employed for the final data presentation and structure plots [57,58].

Elongated thermal ellipsoids and residual electron density peaks near [C7,C9,C10,C12], [C14,C15,C17,C18], and [C20-C23] suggested disorder, which was successfully modeled between two positions each, with independent occupancy ratios. The occupancy ratio values for all the three groups were close and hence were restrained to have the same value for the final least-squares refinement cycles. Appropriate restraints were added to keep the bond distances, angles, and thermal ellipsoids meaningful. H2 and H4 were located from residual electron density peaks and were set riding on the parent oxygen atoms for the final refinement cycles. Remaining hydrogen atoms were geometrically placed and set riding on respective parent atoms.

5. A solution of **6** in hexanes was concentrated by slow evaporation. A colorless plate with very well-defined faces from a representative sample of crystals of the same habit was collected and data were obtained as outlined in Table **S2.** The crystal was kept in a nitrogen stream at T = 99.97(12) K during measurement. Crystal screening, unit cell determination, and data collection were carried out using a XtaLAB Synergy, Dualflex, HyPix diffractometer. The diffraction patterns were indexed and the total number of runs and images were based on strategy calculations from the program CrysAlisPro System [55]. Data were measured using ω scans with Cu K_α radiation. Absorption corrections were applied using CrysAlisPro [55] and the unit cells were refined using the same program System [55]. The structure was solved using ShelXT 2018/2 [56] and refined by full matrix least squares minimization on $\mathbf{F}^2$ using version 2019/1 of ShelXL 2019/1 [56]. All non-hydrogen atoms were refined anisotropically. Hydrogen atom positions were calculated geometrically and refined using the riding model. Olex2 and Mercury were employed for the final data presentation and structure plots [57,58].

The crystals were thin plates. Several batches of crystals were tried which displayed poor diffraction quality. A much smaller plate, which exhibited reasonable diffraction was chosen for collecting the data presented here. Hydrogen atoms attached to O3 and O5 were located from residual electron density peaks and were set riding on the respective parent oxygen atoms with idealized distances. The rest of the hydrogen atoms were placed geometrically, and were set riding. Small residual electron density peaks near O2-O5 suggested minor disorder which was modeled successfully between two positions with an occupancy ratio of 0.95. Appropriate restraints were used to keep the bond distances, angles, and thermal ellipsoids meaningful.

Table S1. Crystallographic data for **2**, **3**, and **4**.

	**2**	**3**	**4**
empirical formula	$C_{20}H_{39}O_5P$	$C_{21}H_{41}O_5P$	$C_{22}H_{43}O_5P$
formula weight	390.48	404.51	418.53
temperature [K]	100.0(4)	100.0(4)	100.03(10)
diffractometer	Rigaku XtaLAB Synergy	Rigaku XtaLAB Synergy	Rigaku XtaLAB Synergy
wavelength [Å]	1.54184	1.54184	1.54184
crystal system	orthorhombic	monoclinic	Monoclinic
space group	$Pna2_1$	$P2_1/c$	$P2_1/c$
unit cell dimensions:			
a [Å]	16.9881(2)	10.89280(10)	10.8749(2)
b [Å]	17.0243(2)	18.88580(10)	18.9166(4)
c [Å]	7.37130(10)	10.93010(10)	11.5913(2)
α [°]	90	90	90
β [°]	90	92.3740(10)	96.138(2)
γ [°]	90	90	90
V [Å^3]	2131.86(5)	2246.60(3)	2370.85(8)
Z	4	4	4
ρ_{calc} [Mg/m^3]	1.217	1.196	1.173
μ [mm^{-1}]	1.355	1.302	1.250
F(000)	856	888	920
crystal size [mm^3]	0.17 × 0.11 × 0.05	0.42 × 0.12 × 0.04	0.09 × 0.06 × 0.02
Θ limit [°]	3.676 to 80.159	4.062 to 74.478	4.089 to 74.504
index range (h, k, l)	–21, 21; –21, 21; –6, 9	–13, 13; –21, 23; –13, 13	–13, 13; –20, 23; –14, 14
reflections collected	21809	40712	24721
independent reflections	3784	4591	4854
R(int)	0.0277	0.0239	0.0387
completeness to Θ	100.00 %	100.00 %	100.00 %
max. and min. transmission	1.000 and 0.755	1.000 and 0.507	0.979 and 0.599
data/restraints/parameters	3784 / 457 / 273	4591 / 467 / 303	4854 / 75 / 312
goodness-of-fit on F^2	1.069	1.051	1.051
R indices (final) [$I > 2\sigma(I)$]			
R_1	0.0329	0.0339	0.0583
wR_2	0.0890	0.0906	0.1553
R indices (all data)			
R_1	0.0333	0.0349	0.0630
wR_2	0.0894	0.0914	0.1590
largest diff. peak and hole [eÅ^{-3}]	0.262 and -0.295	0.327 and -0.288	0.512 and -0.527

Table S2. Crystallographic data for **5** and **6**.

	**5**	**6**
empirical formula	$C_{23}H_{45}O_5P$	$C_{27}H_{53}O_5P$
formula weight	432.56	488.66
temperature [K]	100.00(10)	99.97(12)
diffractometer	Rigaku XtaLAB Synergy	Rigaku XtaLAB Synergy
wavelength [Å]	1.54184	1.54184
crystal system	triclinic	triclinic
space group	*P*-1	*P*-1
unit cell dimensions:		
a [Å]	9.95090(10)	10.3804(3)
b [Å]	10.2240(2)	11.1410(3)
c [Å]	13.5835(2)	12.8308(3)
α [°]	105.2770(10)	79.725(2)
β [°]	111.1350(10)	81.112(2)
γ [°]	96.3110(10)	72.386(3)
V [Å^3]	1210.77(3)	1383.59(7)
Z	2	2
ρ_{calc} [Mg/m^3]	1.186	1.173
μ [mm^{-1}]	1.238	1.136
F(000)	476	540
crystal size [mm^3]	0.53 × 0.39 × 0.08	0.11 × 0.08 × 0.02
Θ limit [°]	3.692 to 74.499	3.521 to 74.494
index range (h, k, l)	−12, 12; −12, 12; −13, 16	−12, 12; −13, 13; −15, 16
reflections collected	45111	27906
independent reflections	4925	5614
R(int)	0.0416	0.0426
completeness to Θ	99.90 %	100.00 %
max. and min. transmission	1.000 and 0.144	1.000 and 0.726
data/restraints/parameters	4925 / 217 / 382	5614 / 153 / 336
goodness-of-fit on F^2	1.046	1.071
R indices (final) [$I > 2\sigma(I)$]		
R_1	0.0497	0.0387
wR_2	0.1376	0.1031
R indices (all data)		
R_1	0.0515	0.0446
wR_2	0.1394	0.1065
largest diff. peak and hole [eÅ^{-3}]	0.638 and -0.437	0.256 and -0.315

II. X-Ray Figures

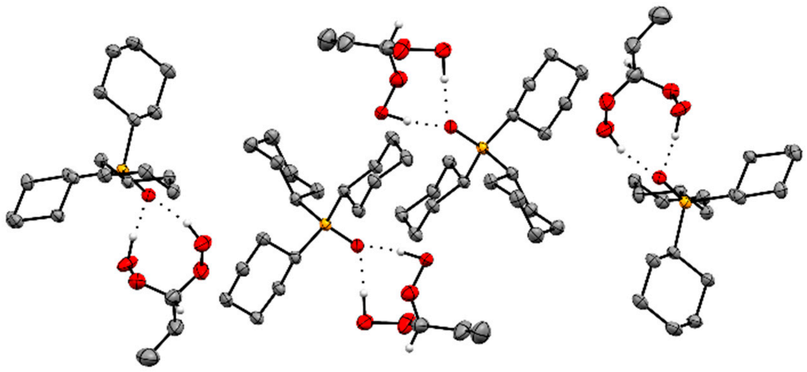

Figure S1. Stacking pattern of two adduct assemblies of $Cy_3PO·(HOO)_2CHCH_2CH_3$ (**3**). Hydrogen atoms except those in $CH(OOH)_2$ moieties are omitted for clarity.

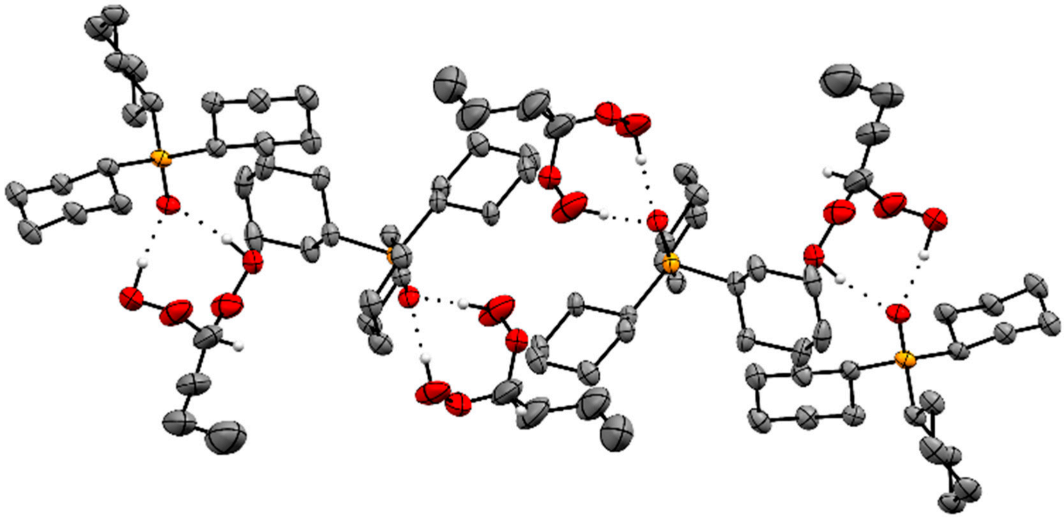

Figure S2. Stacking pattern of four adduct assemblies of $Cy_3PO·(HOO)_2CH(CH_2)_2CH_3$ (**4**). Hydrogen atoms except those in $CH(OOH)_2$ moieties are omitted for clarity.

III. NMR Experiments

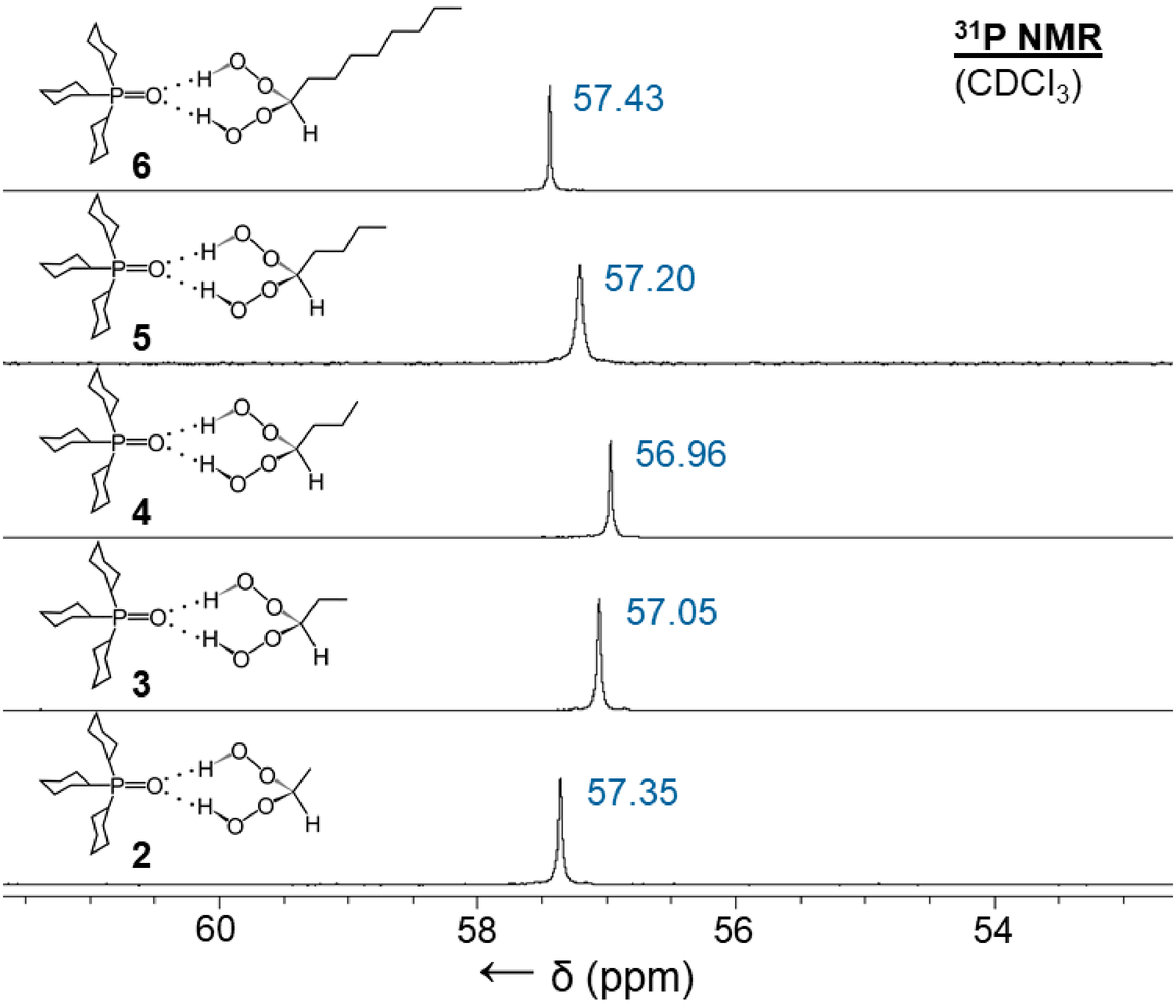

Figure S3. ^{31}P NMR spectra of the adducts **2-6** in $CDCl_3$. All signals are downfield-shifted as compared to the resonance of Cy_3PO ($\delta(^{31}P)$ = 49.91 ppm).

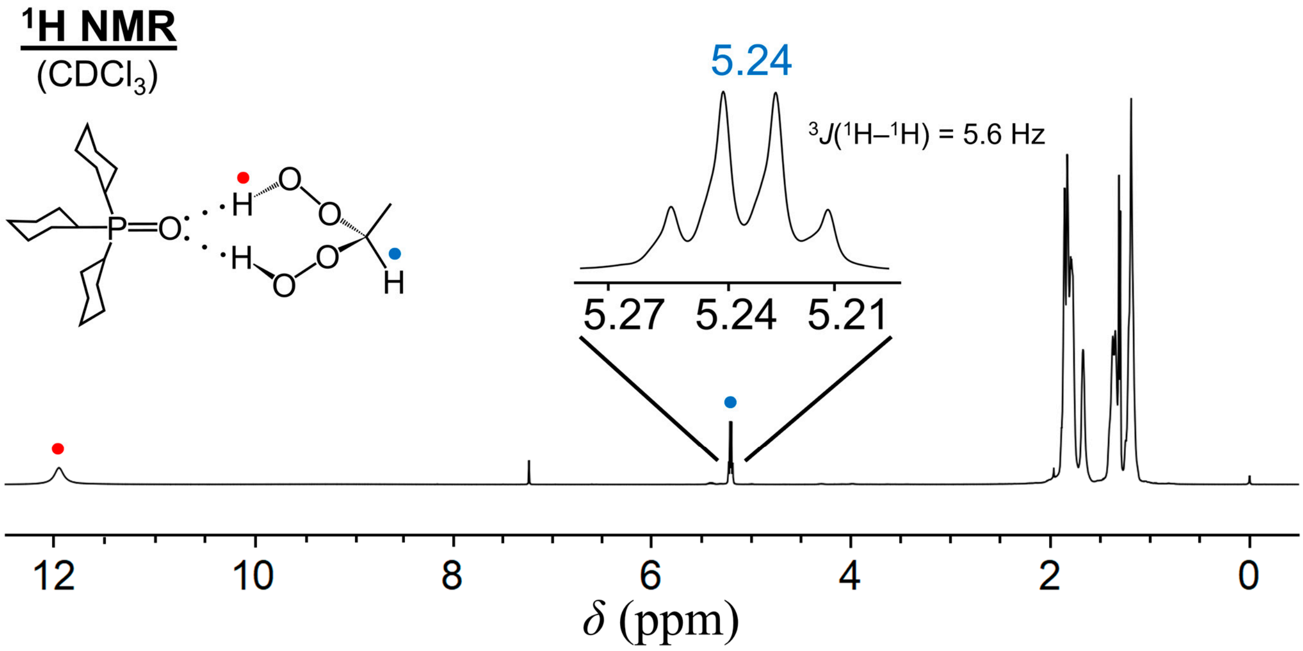

Figure S4. ^{1}H NMR spectrum of the adduct $Cy_3PO{\cdot}(HOO)_2CHCH_3$ (**2**).

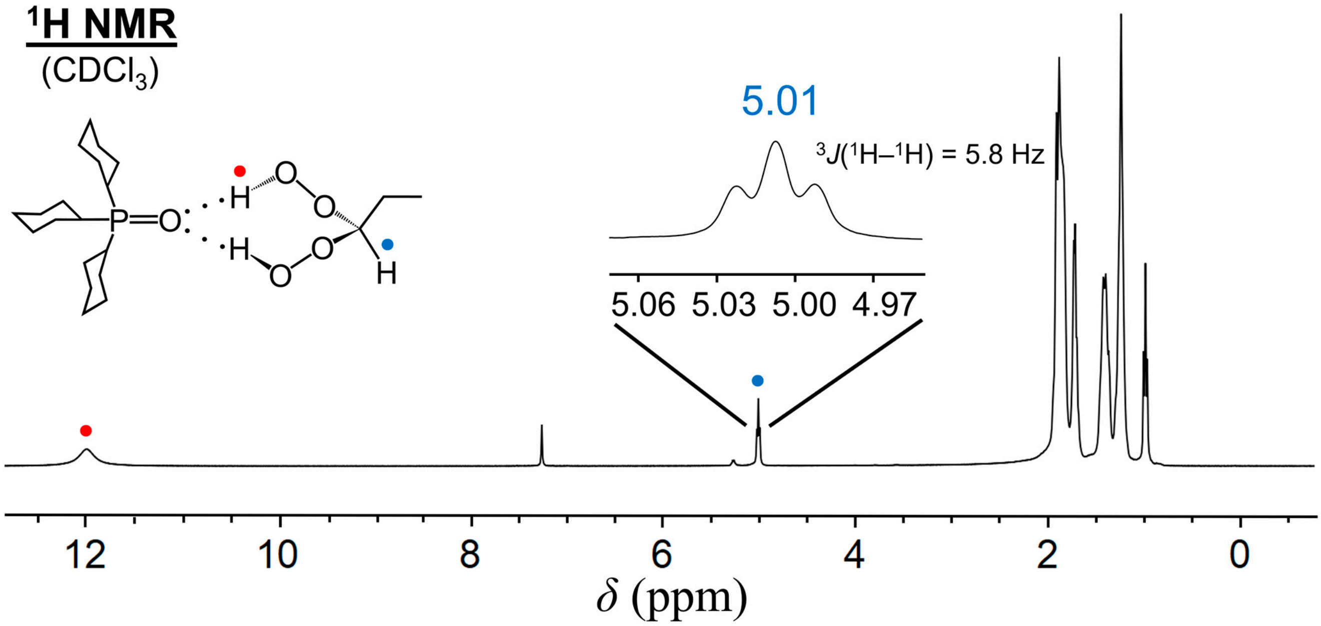

Figure S5. ^{1}H NMR spectrum of the adduct $Cy_3PO{\cdot}(HOO)_2CHCH_2CH_3$ (**3**).

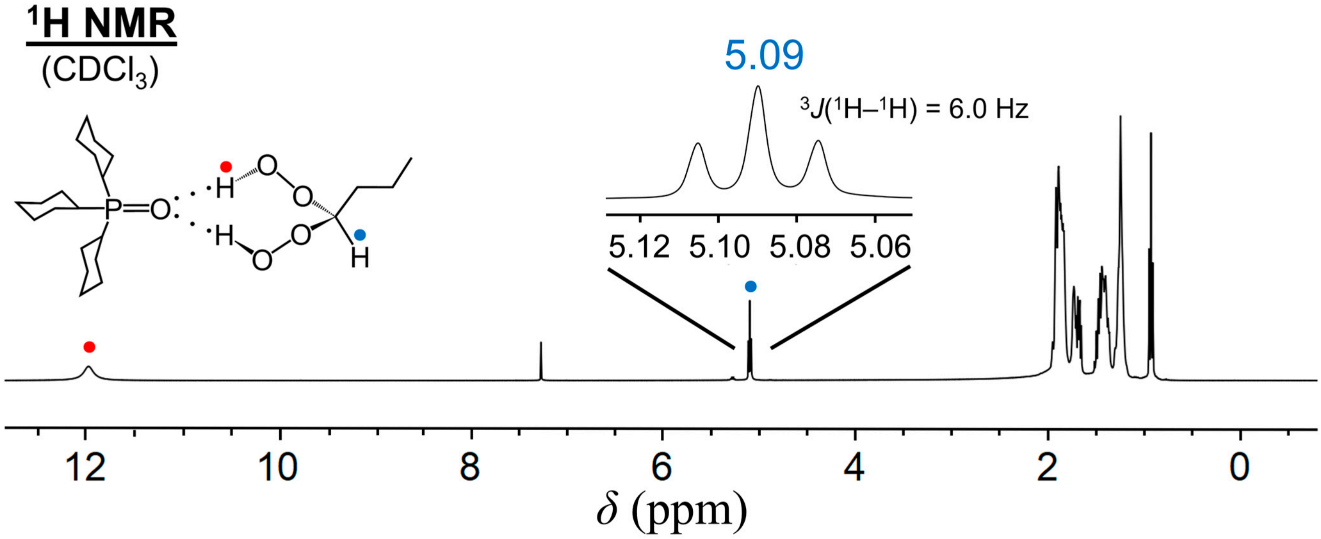

Figure S6. ^{1}H NMR spectrum of the adduct $Cy_3PO{\cdot}(HOO)_2CH(CH_2)_2CH_3$ (**4**).

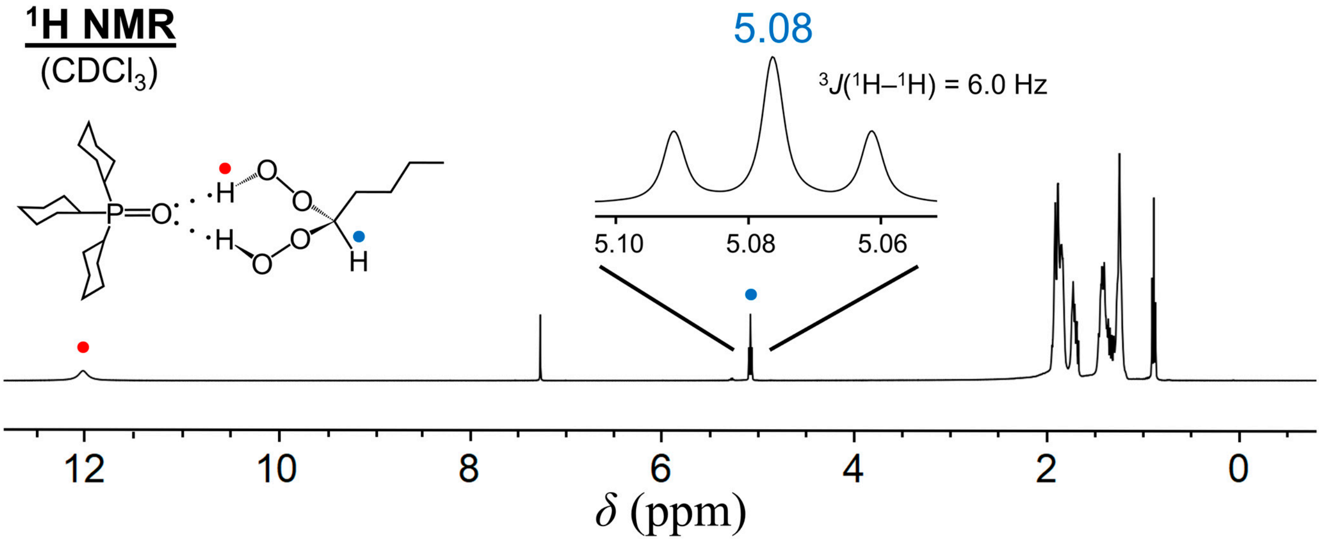

Figure S7. ^{1}H NMR spectrum of the adduct $Cy_3PO \cdot (HOO)_2CH(CH_2)_3CH_3$ (**5**).

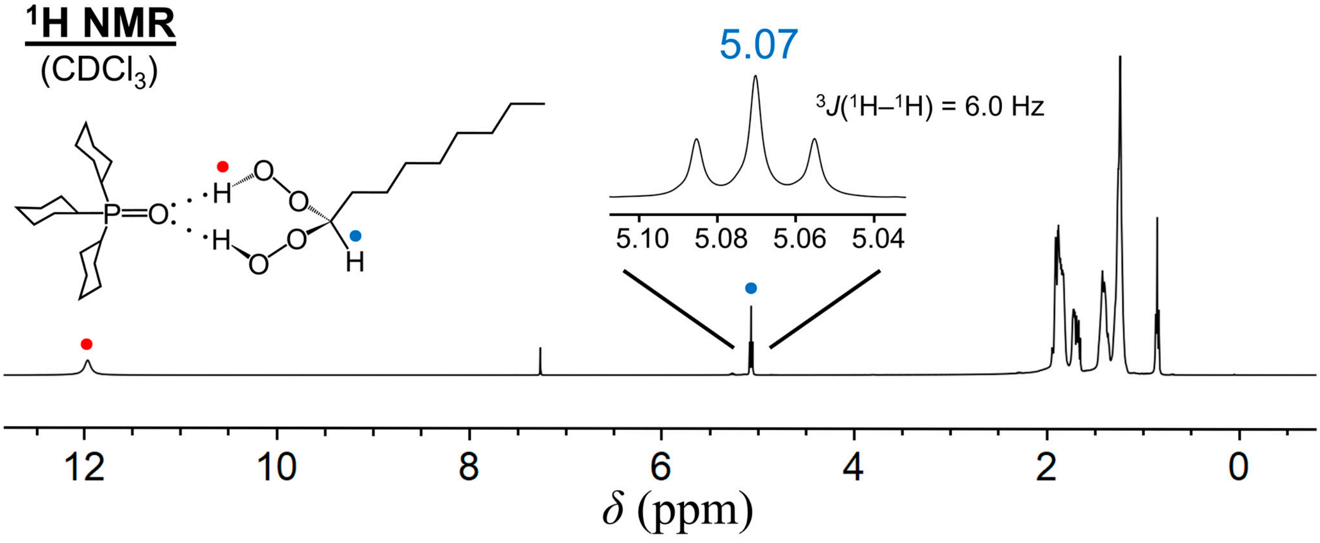

Figure S8. ^{1}H NMR spectrum of the adduct $Cy_3PO \cdot (HOO)_2CH(CH_2)_7CH_3$ (**6**).

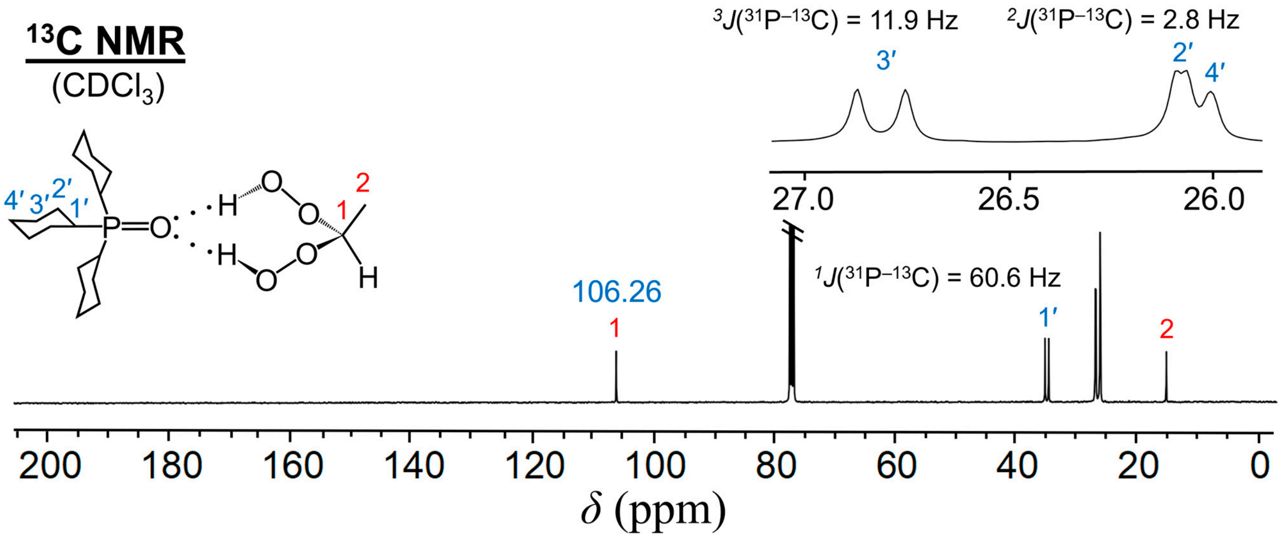

Figure S9. ^{13}C NMR spectrum of the adduct $Cy_3PO{\cdot}(HOO)_2CHCH_3$ (**2**).

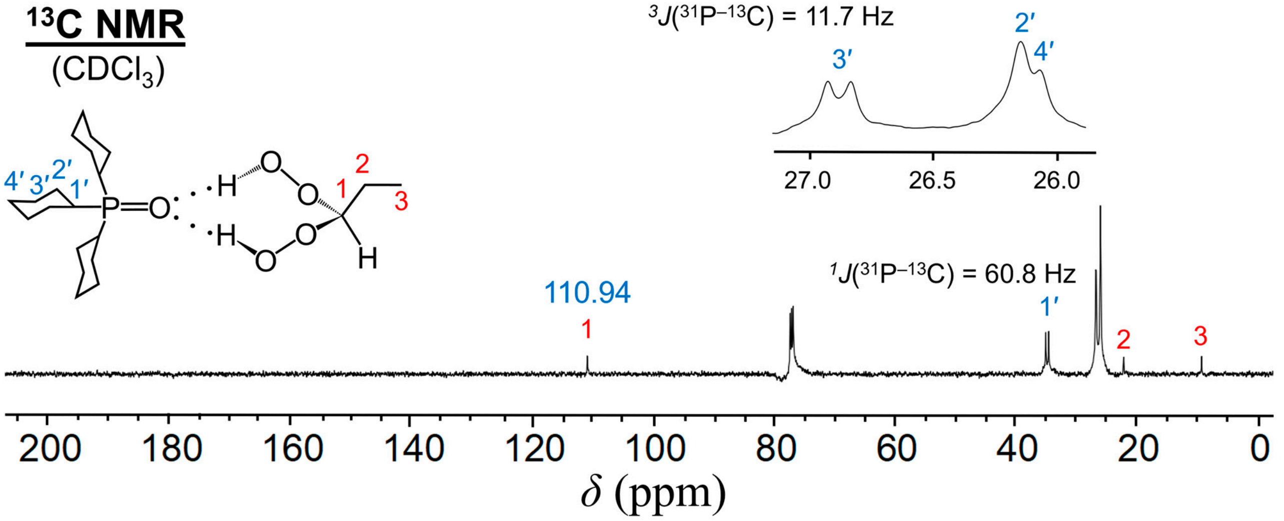

Figure S10. ^{13}C NMR spectrum of the adduct $Cy_3PO{\cdot}(HOO)_2CHCH_2CH_3$ (**3**).

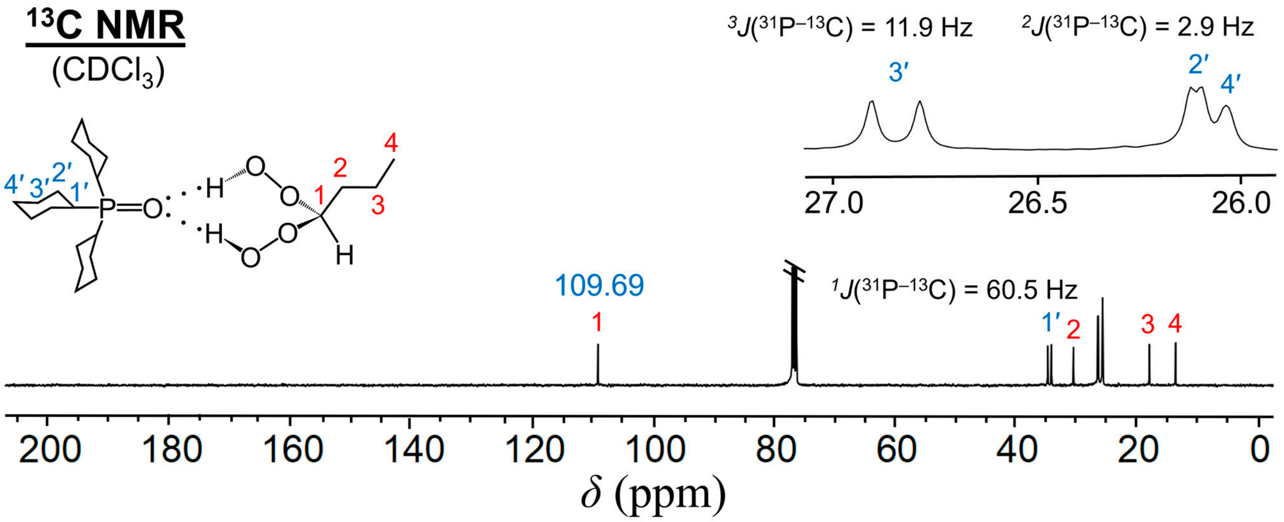

Figure S11. ^{13}C NMR spectrum of the adduct $Cy_3PO{\cdot}(HOO)_2CH(CH_2)_2CH_3$ (**4**).

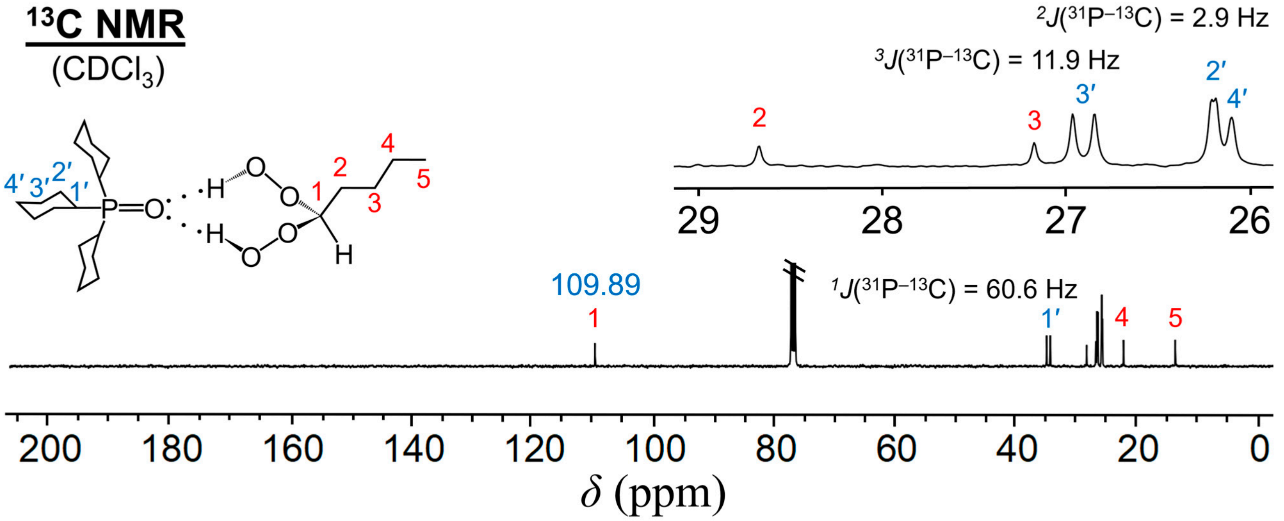

Figure S12. ^{13}C NMR spectrum of the adduct $Cy_3PO{\cdot}(HOO)_2CH(CH_2)_3CH_3$ (**5**).

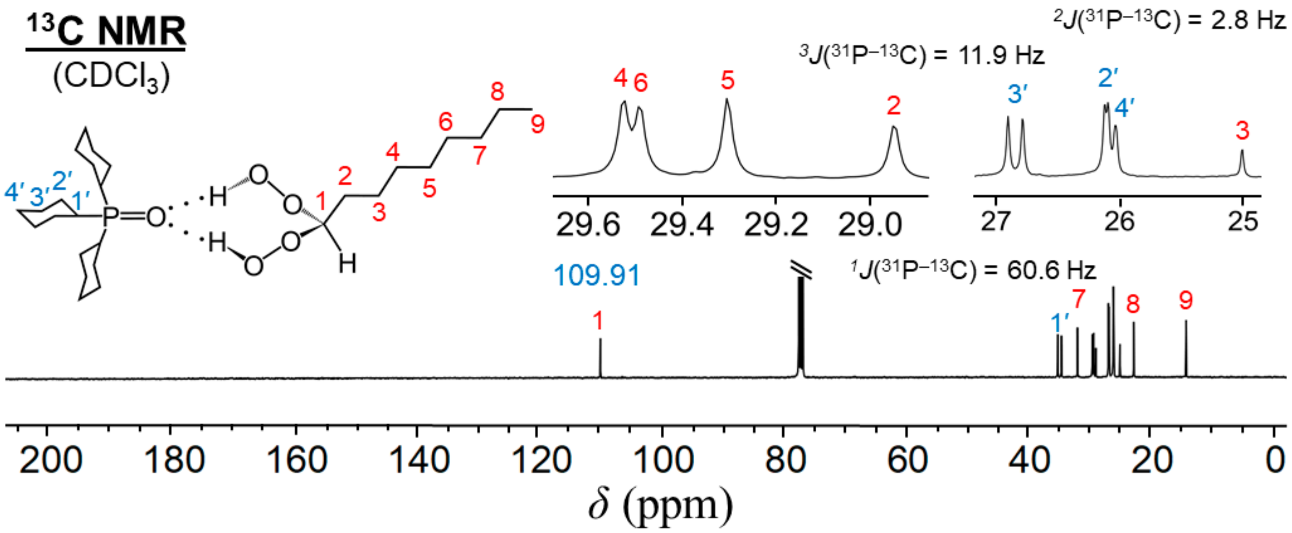

Figure S13. ^{13}C NMR spectrum of the adduct $Cy_3PO{\cdot}(HOO)_2CH(CH_2)_7CH_3$ (**6**). Assignments for C4, C5, and C6 are interchangeable.

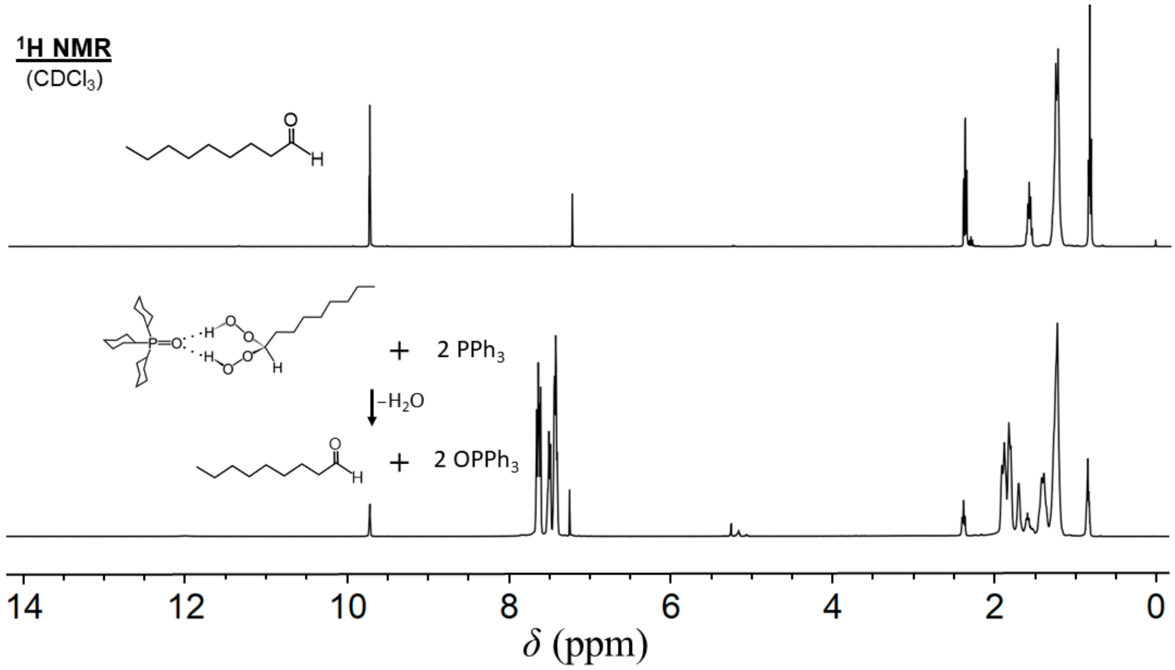

Figure S14. ^{1}H NMR spectra of nonanal (top) and the reaction mixture after combining adduct **6** with 2 equivalents of PPh_3 (bottom).

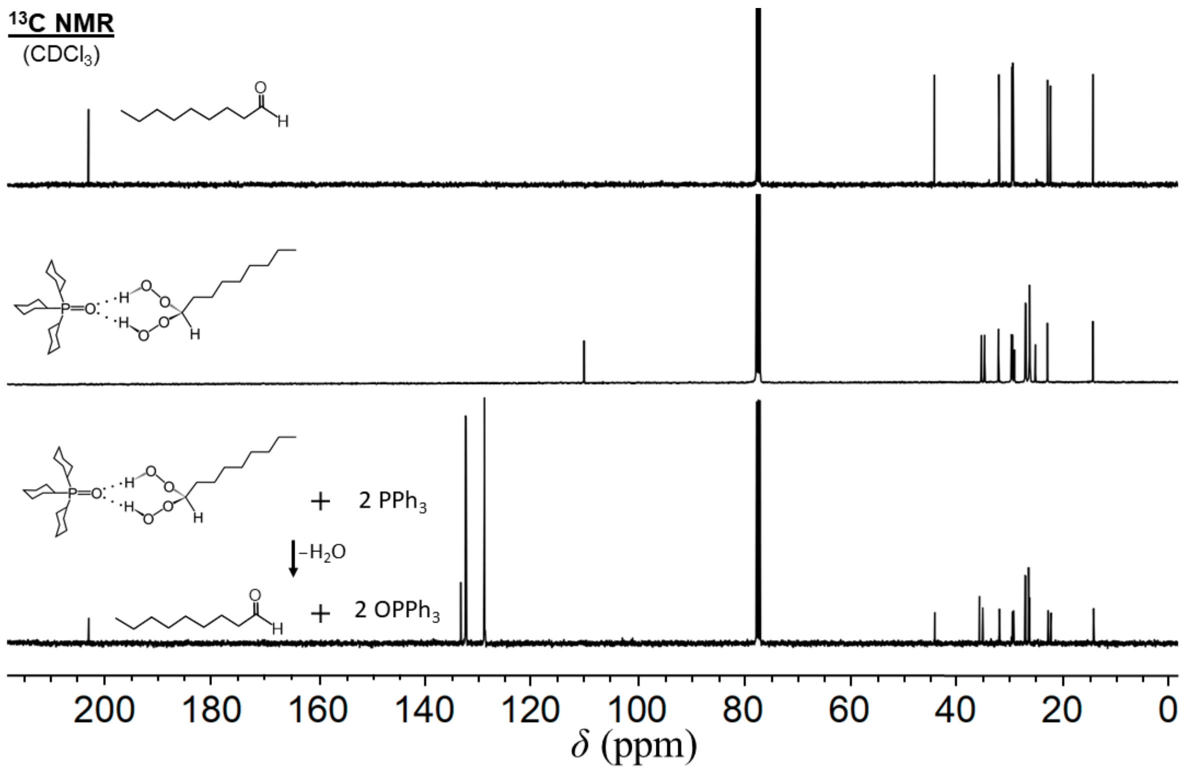

Figure S15. ^{13}C NMR spectra of nonanal (top), the adduct $Cy_3PO{\cdot}(HOO)_2CH(CH_2)_7CH_3$ (**6**) (middle), and the reaction mixture after combining adduct **6** with 2 equivalents of PPh_3 (bottom).
